# Short-chain fatty acids, secondary bile acids and indoles: gut microbial metabolites with effects on enteroendocrine cell function and their potential as therapies for metabolic disease

**DOI:** 10.3389/fendo.2023.1169624

**Published:** 2023-07-25

**Authors:** Karly E. Masse, Van B. Lu

**Affiliations:** Department of Physiology and Pharmacology, University of Western Ontario, London, ON, Canada

**Keywords:** enteroendocrine cells (EEC), gut microbiota, short-chain fatty acids (SCFAs), bile acids (BAs), indoles, glucagon-like peptide-1, signal transduction

## Abstract

The gastrointestinal tract hosts the largest ecosystem of microorganisms in the body. The metabolism of ingested nutrients by gut bacteria produces novel chemical mediators that can influence chemosensory cells lining the gastrointestinal tract. Specifically, hormone-releasing enteroendocrine cells which express a host of receptors activated by these bacterial metabolites. This review will focus on the activation mechanisms of glucagon-like peptide-1 releasing enteroendocrine cells by the three main bacterial metabolites produced in the gut: short-chain fatty acids, secondary bile acids and indoles. Given the importance of enteroendocrine cells in regulating glucose homeostasis and food intake, we will also discuss therapies based on these bacterial metabolites used in the treatment of metabolic diseases such as diabetes and obesity. Elucidating the mechanisms gut bacteria can influence cellular function in the host will advance our understanding of this fundamental symbiotic relationship and unlock the potential of harnessing these pathways to improve human health.

## 1 Introduction

**Figure 1:**
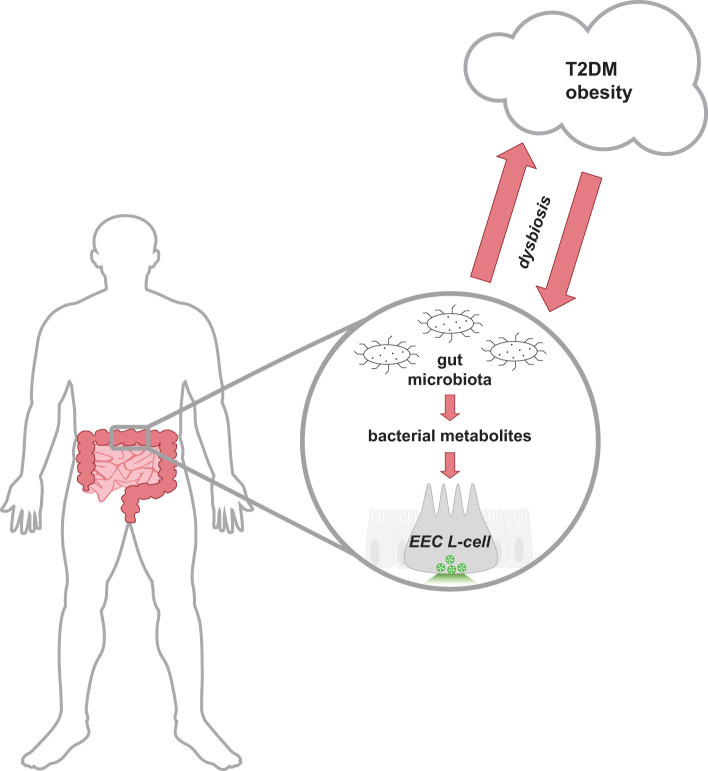
Schematic representation of the bidirectional relationship between the host-gut microbiota equilibrium and metabolic health. Cells lining the distal gastrointestinal tract are in direct contact with bacterial metabolites produced by the gut microbiota, and thus can contribute to host health. In a healthy state, the gut microbiota produces metabolites that activate receptors on distal EECs to mediate insulinotropic effects by the release of secretory vesicles containing GLP-1. Gut dysbiosis alters the intestinal composition and metabolites produced and is associated with the development of T2DM and obese-related diseases. Research has proposed that the dysregulation of metabolism in metabolic diseases releases molecules that can reduce the abundance of intestinal bacteria and alter the function of the ecosystem. EEC, enteroendocrine cell; GLP-1, glucagon-like peptide-1; T2DM, type 2 diabetes mellitus.

The gut microbiota encompasses all the microorganisms (bacteria, fungi, archaea, viruses) that have colonized the gastrointestinal tract of host animals. The gut microbiota is composed of trillions of microbes, with current estimates suggesting the collective genome of gut bacteria outnumbers the host human genome over 1000:1 (1). Bacteria residing in the gut predominantly belong to the phyla *Bacillota* (also known as *Firmicutes*) and *Bacteriodota* (aka *Bacteroidetes*); however, bacteria belonging to *Actinomycetota* (aka *Actinobacteria*)*, Pseudomonadota* (aka *Proteobacteria*), or *Verrucomicrobiota* (aka *Verrucomicrobia*) are also represented (2). This complex and diverse environment of microorganisms contributes to a symbiotic relationship with the host, assisting in host physiological functions such as nutrient and energy metabolism, maintenance of intestinal barrier integrity, and immune protection (3–5). Changes in gut microbiota populations have been associated with a multitude of human disease states, including the metabolic diseases Type 2 diabetes mellitus (T2DM) and obesity (**Figure 1**). Reduced bacterial diversity and richness have been reported in human and animal models of obesity and diabetes (6–8). Dysbiosis of host-gut microbiota equilibrium may precede metabolic disease as similar shifts in intestinal gut bacteria composition can disrupt nutrient and energy metabolism (9). Due to the global health burden of metabolic diseases, there is great interest in developing novel therapeutic approaches including targeting mechanisms involving the gut microbiota.

Diet is a key factor in metabolic health and can influence the progression of metabolic disease. It can also regulate gut microbiota health as resident gut bacteria metabolize host-digested macronutrients to produce an additional class of active biomolecules. For instance, complex carbohydrates undergo bacterial fermentation to produce short-chain fatty acids (SCFAs) (10–12) and the amino acid tryptophan is further metabolized by gut bacteria to produce indole and other indole-derivatives (13, 14). Cholesterol-derived bile acids released from hepatocytes are also modified by gut bacteria to improve solubility and facilitate recycling of bile acids in the distal colon (15). These bacterial metabolites themselves may mediate the effects of gut microbiota on host health as changes in the levels of SCFAs, indoles, and secondary bile acids are associated with metabolic disease (16–18) and restoration of levels can attenuate disease progression and severity (19–24). Although many studies have carefully identified and quantified the levels of bacterial metabolites produced in humans (10, 25–27), the signaling pathways mediating the cross-talk between microbiota-derived metabolites and host physiology has yet to be fully elucidated.

A specialized population of intestinal epithelial cells called enteroendocrine cells (EECs) are strategically positioned to mediate the effects of bacterial metabolites on host health. EECs have an open-type morphology that spans the intestinal epithelial cell layer. The apical cell side faces the luminal interface with microvilli-like structures that are exposed to nutrients and bacterial metabolites. EECs also express several different types of nutrient-sensitive receptors (28) that facilitate their role as intestinal chemosensors. The basolateral cell side of EECs connects the release of hormones to the intestinal circulatory system. The gut hormone released can exert effects on host physiology thereby providing a mechanistic link between bacterial metabolism of nutrients and host health. The gut hormone glucagon-like peptide-1 (GLP-1), secreted from a subset of EECs called L-cells, is of interest in the context of metabolic disease because of GLP-1’s anorexigenic and hypoglycemic properties (29). GLP-1 mimetics have been used for the treatment of obesity and T2DM (30–35). Furthermore, the mechanism of improved metabolic status following bariatric surgery has been attributed to enhanced GLP-1 release (36–38). EEC L-cells also secrete peptide-YY (PYY), a gut hormone involved in appetite regulation (39, 40). Interestingly, the distal small intestine and colon harbor the greatest density of PYY and GLP-1 releasing L-cells (41), paralleling the distribution of gut bacteria (42). Thus, studying bacterial metabolite sensing in EEC L-cells can advance our understanding of the mechanisms by which gut microbiota regulate host metabolic health. It can also provide a novel therapeutic avenue for the treatment and management of metabolic disease.

The focus of this review will be gut microbiota-derived metabolites that are most abundant in the human colon, specifically SCFAs, secondary bile acids and indoles, and how each bacterial metabolite modulate EEC L-cell function (43–47). We will detail the signaling pathways that are recruited in EEC L-cells following exposure to each bacterial metabolite. In addition, we will describe how the levels of these bacterial metabolites are altered during metabolic disease and discuss therapeutic approaches that target these bacterial metabolite signaling pathways.

## 2 Metabolic products of microbes

### 2.1 Production of short-chain fatty acids by the gut microbiota

SCFAs are monocarboxylic acids of 1-5 carbon chain lengths and are the most abundant bacterial metabolite produced in the gut (11, 12). The majority of bacterial SCFAs synthesized (>95%) include acetate (C2), propionate (C3), and butyrate (C4) in a molar ratio of approximately 3:1:1, respectively (10, 48). Production of SCFAs is most abundant in the caecum and ascending limb of the colon in humans (>100 mM) (10). In humans, undigested fiber passes through the small intestine largely unabsorbed before entry into the colon and metabolism by both Gram-negative and Gram-positive bacteria. The production of the smaller chained SCFAs acetate and propionate are favored by *Bacteroidota*, whereas *Bacillota* primarily produce butyrate as a metabolic product (49). Bacterial fermentation of indigestible carbohydrates yields the majority of SCFAs produced, but a small fraction of SCFAs produced (1%) stem from bacterial metabolism of dietary amino acids (50). Notably, the liver can generate significant levels of acetate (~1 mM) during bouts of chronic alcohol consumption that can enter circulation and impact gastrointestinal function (51).

**Figure 2:**
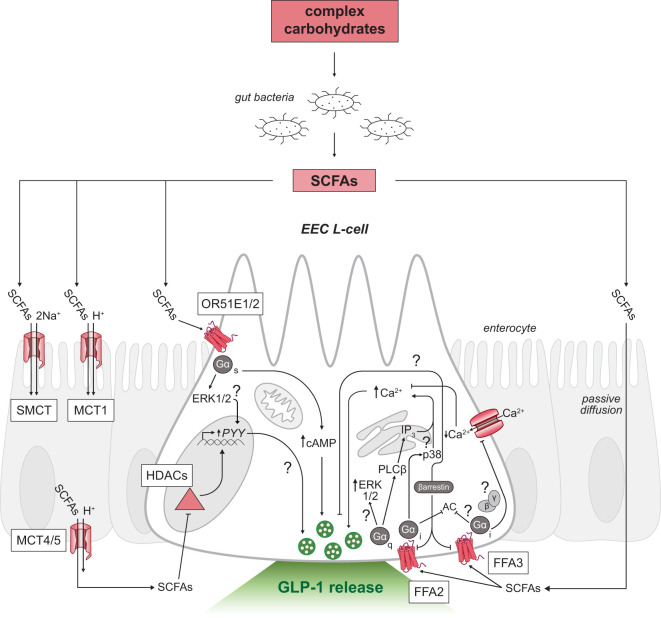
SCFA-triggered intracellular signaling mechanisms in GLP-1 releasing EEC L-cells. Schematic of an L-cell (white) surrounded by enterocytes (grey). Complex carbohydrates are substrates for resident gut bacteria to produce SCFAs in the distal gastrointestinal tract. SCFAs can signal through multiple receptors on both the apical (top) and basolateral membranes (bottom) of L-cells. Uptake of SCFAs by SMCT and MCT across the intestinal epithelium to the basolateral side shown in enterocytes. SCFAs inhibit HDACs or activate G-protein coupled receptors FFA2, FFA3 and OR51E1/2 in L-cells. Unresolved mechanisms are marked with a question mark. EEC, enteroendocrine cell; SCFAs, short-chain fatty acids; FFA2/3, free fatty acid receptor 2 or 3; OR51E1/2, olfactory receptor subfamily 5E1 or 2; GLP-1, glucagon-like peptide-1; Ca^2+^, calcium ions; SMCT, sodium-dependent monocarboxylate transporter; MCT, H^+^-dependent monocarboxylate transporter; Na^+^, sodium ions; HDACs, histone deacetylases; AC, adenylyl cyclase; cAMP, cyclic adenosine monophosphate; PLCβ, phospholipase C beta; ERK1/2, extracellular signal-regulated kinases; IP_3_, inositol triphosphate.

Following production, SCFAs are almost exclusively taken up by colonocytes *via* H^+^-dependent or sodium-dependent monocarboxylate transporters (MCTs and SMCTs, respectively; **Figure 2**) (52). Colonic absorption of SCFAs accounts for approximately 5-10% of the body’s total energy requirement, with butyrate acting as the predominant source of energy (53). The absorbed butyrate is largely utilized by colonocytes for energy, and the remaining absorbed SCFAs are transported through portal blood circulation back to the liver where SCFAs are primarily converted to glucose stores (4). Therefore, the levels of SCFAs that reach systemic circulation are much lower in concentration than the levels measured in the colon (10, 54).

### 2.2 Metabolism of bile acids by the gut microbiota

**Figure 3:**
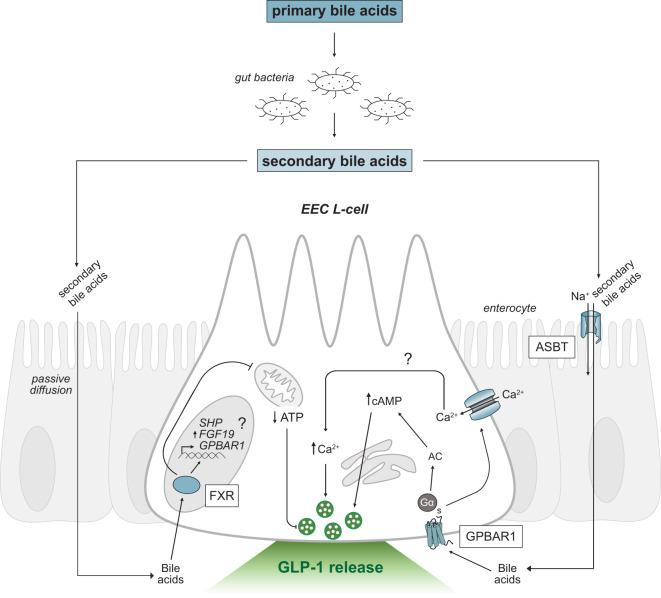
Secondary bile acid-triggered intracellular signaling mechanisms in GLP-1 releasing EEC L-cells. Schematic as in **Figure 2**. Primary bile acids are converted to secondary bile acids by intestinal gut bacteria. Secondary bile acids passively diffuse or are transported across the intestinal epithelium *via* ASBT and activate the G-protein coupled receptor, GPBAR1 and nuclear receptor, FXR. Activation of GPBAR1 by secondary bile acids results in GLP-1 secretion. FXR regulates *SHP* and *FGF19* expression and may regulate *GPBAR1* gene transcription (marked by a question mark). EEC, enteroendocrine cell; GPBAR1, G-protein coupled bile acid receptor; FXR, Farnesoid-X receptor; GLP-1, glucagon-like peptide-1; ASBT, apical sodium-dependent bile acid transporter; Ca^2+^, calcium ions; AC, adenylyl cyclase; cAMP, cyclic adenosine monophosphate; ATP, adenosine triphosphate; SHP, short heterodimer protein; FGF19, fibroblast growth factor 19; Na^+^, sodium ions.

Bile acids are the primary metabolic end products of cholesterol catabolism (55, 56) and account for the majority of cholesterol turnover in humans. Hydroxylation and modification of cholesterol in the liver generates the primary bile acids, cholic acid (CA) and chenodeoxycholic acid (CDCA) in humans (57), and CA and muricholic acid (MCA) in rodents (58). Most primary bile acids are conjugated with glycine or taurine, to increase solubility properties, and are stored in the gallbladder (56). Conjugated bile acids comprise the majority of secreted bile; however, phospholipids, cholesterol, exogenous drugs, and environmental toxins contribute a small component (59). Following the consumption of fat, the gut hormone cholecystokinin is released which stimulates the contraction of the gallbladder to release bile acids into the proximal small intestine. Bile acids act as powerful detergent molecules, forming solubilizing micelles that promote the digestion and absorption of dietary lipids and fat-soluble vitamins (59). The total levels of bile acids in the enterohepatic circulation, or bile acid pool, remains consistent due to highly efficient (95%) reabsorption of bile acids in the small intestine (55). Conjugated primary bile acids are actively reabsorbed in the distal ileum *via* the apical sodium-dependent bile acid transporter (ASBT; also known as the ileal sodium-dependent bile acid transporter, IBAT; **Figure 3**), whereas unconjugated bile acids can passively diffuse through enterocytes. The reabsorbed bile acids are shuttled across enterocytes to the basolateral membrane and are recycled back to the liver through portal blood circulation. Conjugated bile acids are taken up by hepatocytes *via* the sodium taurocholate cotransporting polypeptide (NTCP) while unconjugated bile acids are taken up by the organic anion transporting polypeptide (OATP), which is also responsible for uptake of bilirubin (55, 59). The bile acid pool is tightly regulated through the coordination between synthesis, reabsorption, and excretion of bile acids by the liver.

The remaining bile acids that escape absorption in the small intestine (5%) act as substrates for anaerobic metabolism in the colon (15, 60) or are excreted with feces. Bile salt hydrolase (BSH), produced by intestinal bacteria, converts conjugated primary bile acids to secondary bile acids through a series of biotransformation reactions, thus increasing the diversity of bile acids. In humans, deoxycholic acid (DCA) and lithocholic acid (LCA) are the predominant secondary bile acids produced (15, 57), whereas in rodents the predominant secondary bile acids generated are DCA and ω-MCA (58). At the phyla level, bacterial populations encoding BSH, such as *Bacillota, Bacteroidota* and *Actinomycetota*, have been shown to play an important role in the production of secondary bile acids (61). Other human intestinal archaea species, *Methanobrevibacter smithii* and *Methanosphera stadmanae*, also encode for BSH and can contribute to the production of secondary bile acids (62). Other bile acid transformations catalyzed by bacterial enzymes include the actions of hydroxysteroid dehydrogenases (HSDs), which alters the hydrophobicity and toxicity of bile acids (15).

### 2.3 Metabolism of tryptophan by the gut microbiota

Tryptophan is an essential aromatic amino acid that must be consumed as the body lacks the enzymes necessary to synthesize tryptophan. Following protein digestion, most of the liberated tryptophan is absorbed in the small intestine and endogenously metabolized: up to 95% of ingested tryptophan is converted to kynurenic acid or nicotinamide adenine dinucleotide (NAD^+^) (63, 64) *via* the kynurenine pathway, and 1-2% of ingested tryptophan is converted to serotonin *via* tryptophan hydroxylase 1 activity (65, 66). The remaining ingested tryptophan that escapes absorption (4-6%) enters the colon and is metabolized by intestinal bacteria (13). More than 85 different Gram-positive and Gram-negative bacterial species express tryptophanase (67), the enzyme that catalyzes the hydrolytic β-elimination of tryptophan to indole, pyruvate, and ammonia (68). Indole production also depends on the tryptophan-specific transporter, TnaB, expressed in bacteria to facilitate tryptophan uptake. Other transporters such as AroP and Mtr permeases may also facilitate bacterial uptake of tryptophan (69). Indole is the most abundant bacterial metabolite of tryptophan degradation produced with the average physiological concentration between 0.25-1.1 mM in human feces (14). Bacterial metabolism of tryptophan can also give rise to other indole-moiety containing derivatives. Indole-3-acetic acid (IAA) is an intermediate formed during a series of decarboxylation reactions from indole-3-pyruvic acid (IPyA). IAA can be further catabolized to indole-3-aldehyde (IAld) and 3-methylindole (skatole). Alternatively, bacterial enzymes catalyze reduction and dehydration reactions to produce indole-3-propionic acid (IPA). Physiological levels of IPA range between 1-10 µM in human serum (70). Another bacterial transformation of tryptophan can occur through the actions of tryptophan decarboxylases to produce tryptamine (71). Bacteria also express decarboxylases to convert indole to tryptamine (72).

**Figure 4:**
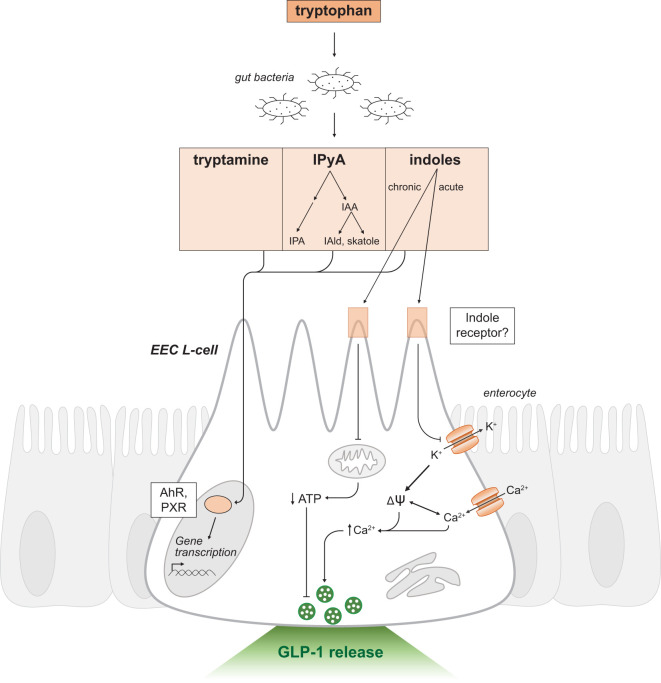
Indole-triggered intracellular signaling mechanisms in GLP-1 releasing EEC L-cells. Schematic as in **Figure 2**. Tryptophan is a substrate for resident gut bacteria to produce the metabolites tryptamine, IPyA and indoles. IPyA is a precursor to the metabolites IAA, IAld, skatole and IPA. Indole and other metabolites passively diffuse across the intestinal epithelium and activate the transcription factors AhR and PXR to regulate gene transcription. Indole may mediate effects through a yet to be determined receptor. There are dual and opposing effects on GLP-1 release by acute and chronic exposure to indoles. Acute indole stimulates release of secretory vesicles containing GLP-1 by a voltage-gated potassium channel blockade. Chronic indole exposure suppresses mitochondrial activity to produce ATP. IPyA, indole-3-pyruvic acid; IAA, indole-3-acetic acid; IAld, indole-3-aldehyde; IPA, indole-3-propionic acid; EEC, enteroendocrine cell; AhR, Aryl hydrocarbon receptor; PXR, Pregnane-X-receptor; ψ, membrane potential; K^+^, potassium ions; Ca^2+^, calcium ions; ATP, adenosine triphosphate.

Following production, indole and other metabolic derivatives can passively diffuse through the plasma membrane to exert intracellular effects on intestinal epithelial cells (**Figure 4**). Metabolites may also enter enterohepatic circulation and undergo further oxidative metabolism by cytochrome P450 (CYP450) or detoxification by enzymes in the liver (73). For instance, indole undergoes sulfation in the liver to produce indoxyl sulfate, a uremic toxin which accumulates during renal insufficiency inducing fibrosis in damaged proximal tubule cells (74). Alternatively, indole may be reabsorbed passively or actively across bacterial membranes and activate a variety of bacterial processes (75–77).

## 3 Signaling mechanisms

Multiple intracellular signaling pathways have been implicated in gut hormone secretion with concurrent recruitment of several different pathways suggested to be necessary to stimulate release from EECs (78, 79). This section examines the various signaling pathways activated by bacterial metabolites highlighted in this review, and their known effects on GLP-1 release from EEC L-cells.

### 3.1 Intracellular signaling pathways in EEC L-cells activated by SCFAs

In addition to acting as a local energy source for colonocytes, SCFAs may signal through cell surface G-protein coupled receptors (GPCRs) that activate a series of intracellular effector molecules to produce various physiological responses (**Figure 2**). SCFAs activate several GPCRs including: the free fatty acid receptor 2 and 3 (FFA2 and FFA3) (80, 81), the olfactory receptor subfamily 51E1 and 51E2 (OR51E1 in human/Olfr558 in mouse; OR51E2 in human/Olfr78 in mouse) (82), and the hydroxycarboxylic acid receptor, HCAR2 (also known as the G-protein coupled receptor 109A (GPR109A) or the niacin receptor) (83). SCFAs can also affect gene expression through the inhibition of histone deacetylases (HDACs) (84). EECs express HDACs and SCFA-responsive GPCRs (85–89), except HCAR2 which is primarily localized in adipose and immune tissue (83).

The most potent endogenous ligands of FFA2 identified thus far are acetate and propionate (80, 81). To a weaker extent, butyrate can also stimulate the receptor. *Ffar2* expression increases along the longitudinal axis of the gastrointestinal tract, with highest expression in the distal ileum and colon (90). *Ffar2* expression was also found in leukocytes below the epithelial layer using an *Ffar2-*reporter mouse (88) and in the colonic epithelial layer of rats (90) and humans (89) by immunohistochemistry analysis.

Activation of FFA2 couples intracellularly through the G-protein families, Gα_q_ and Gα_i_ (91–93). Recruitment of the Gα_q_-coupled FFA2 signaling pathway in intestinal murine L-cells triggered SCFA-mediated GLP-1 secretion by promoting activity of phospholipase C-dependent production of inositol triphosphate (IP_3_), thereby increasing mobilization of calcium from intracellular stores (88, 92). GLP-1 release was found to be stimulatory in the presence of SCFAs with murine cell lines (94, 95) and murine primary colonic cultures, an effect attributed to FFA2- and FFA3-dependent mechanisms (92, 95). Compound 1, a selective FFA2 agonist, stimulated GLP-1 secretion by FFA2 (96) and this effect was lost in the presence of a Gα_q_ inhibitor, FR900359 (91), thereby supporting Gα_q_-mediated FFA2 signaling mechanisms. Furthermore, pertussis toxin, a Gα_i_-protein uncoupler was shown to not be involved in SCFA-triggered GLP-1 release nor was the Gα_i_-biased FFA2 ligand, AZ1729 (91).

Recruitment of Gα_i_-coupled FFA2 remains unclear. Canonical Gα_i_-signaling mechanisms decrease cyclic adenosine monophosphate (cAMP) production by inhibiting adenylyl cyclase and thus, hormone secretion. However, in duodenal STC-1 and primary colonic cultures, propionate promoted GLP-1 secretion *via* Gα_i-_coupled FFA2 activation and downstream phosphorylation of a class of mitogen-activated protein kinases, p38 (97). This study suggests a spatial discrimination between the pleiotropic actions of the FFA2 receptor as Gα_q_-mediated FFA2 signaling occurred at the cell membrane and Gα_i_ signaling was internalized, thus diversifying the downstream effector molecules activated in EECs (97). A possible convergence of downstream signaling pathways involving the phosphorylation of extracellular signal-regulated kinases (ERK) is possible as both Gα_q_- and Gα_i_-coupled signaling pathways can activate this effector molecule (94). Future research into how Gα_i_-coupled FFA2 is affected in other *in vitro* and *in vivo* L-cell models is warranted to confirm signaling through p38.

In addition, FFA2 is postulated to recruit β-arrestin, a protein involved in the downregulation of GPCRs (98). FFA2 has been shown to employ β-arrestin dependent signaling for transcriptional regulation of proinflammatory cytokine expression *in vitro* (99). In murine and human overexpression studies, agonist stimulation of FFA2 supported β-arrestin recruitment (94) suggesting a possible signaling pathway mediated in L-cells. FFA2 may have the capacity to employ different effector molecules depending on the spatial and temporal gradients of the receptor and needs of the host. Further studies are needed to investigate the functional selectivity of FFA2 under different metabolic conditions.

FFA3 is another free fatty acid receptor responsive to SCFAs that preferentially binds to the SCFAs propionate, butyrate and valerate (80, 81). Mouse *Ffar3* expression paralleled the expression of glucagon (*Gcg*, the gene that encodes for GLP-1), with high transcript levels found in the distal small intestine and colon (92). EEC expression of *Ffar3* was confirmed in a reporter mouse model (88). Additional *Ffar3* expression was described in enteric neurons and vagal afferent neurons that innervate the gastrointestinal tract (100). However, other studies using *in situ* hybridization failed to detect *Ffar3* expression in the nodose ganglion, but rather found expression in sympathetic ganglia innervating the intestines (101). Interestingly, in epithelial cells of the human colonic mucosa, Tazoe et al. (2009), demonstrated co-localization of FFA3 with PYY, the gut hormone co-localized with GLP-1. Co-localization was not observed between FFA3 and serotonin, a marker of another EEC population the enterochromaffin cells (102).

FFA3 exclusively recruits G-proteins of the Gα_i_ family (93), but the cellular mechanism of FFA3 signaling has yet to be fully demonstrated. A study found that the selective FFA3 agonist, AR420626, promoted GLP-1 release from primary colonic cultures (88) and perfused intact colons (103). However, FFA3 activation in sympathetic neurons has been shown to inhibit voltage-gated calcium channels through a Gβγ-mediated mechanism (101), thus inhibiting neurotransmitter release. Similarly, β-arrestin may also be involved in FFA3 activation. In monocytes, FFA3 activation increased intracellular calcium signaling and recruited β-arrestin 2 (104), though, the involvement of β-arrestin in an EEC system has yet to be determined. Indeed, SCFA-triggered FFA3 signaling warrants further investigation.

It is possible that other SCFA-responsive receptors could be involved in the outcome of GLP-1 secretion in L-cells. Originally discovered in olfactory epithelium, the olfactory receptor subfamily 51E1/2 (OR51E1/2; Olfr558/Olfr78 in mouse) are other receptors responsive to SCFAs (82). Acetate and propionate, but not butyrate, are potent endogenous ligands for these receptors (87, 105). Expression of Olfr78 is localized in murine EECs of the colon, especially PYY-positive cells (87) and serotonin-producing enterochromaffin cells (106), though the function of the receptor remains unclear. Expression of the OR51E1 in a human L-cell line NCI-H716 was demonstrated, as well as stimulated GLP-1 secretion following selective receptor activation (107). The mechanism of enhanced GLP-1 secretion involved an increase in intracellular cAMP and phosphorylated ERK (107). Moreover, OR51E1 knockdown reduced GLP-1 secretion, supporting the receptor’s role in mediating the effects of SCFAs on EEC L-cells (107).

In addition to activating cell membrane receptors, SCFAs exert genomic effects by the inhibition of HDACs. Activation of HDACs modifies chromatin structure by removing an acetyl group from histone proteins which reduces DNA accessibility to transcriptional activity. HDAC activity has been implicated in gut development (108) and immune tissue regulation (85, 109). In the colon, HDACs are inhibited by both butyrate and propionate (85, 86), though butyrate is the most effective inhibitor of HDACs (110, 111). This is supported by previous studies suggesting that HDAC inhibition by butyrate induces expression of many genes in various tissues and cell lines (109, 112). Understanding HDAC-mediated changes in expression is physiologically relevant as diets high in fiber results in chronic elevation of SCFA levels, which can lead to lasting changes in gut function. For instance, colonic *Gcg* expression was increased in rats on a fiber-rich diet compared with animals on a chow-fed diet (113). SCFAs have also increased the number of L-cells in the intestinal epithelium and increased endogenous secretion of GLP-1 in both mouse and human organoids *in vitro* (114). As further support, there is a reduction of GLP-1 releasing L-cells in germ-free mice lacking intestinal microbiota (115, 116). However, in cell line models of human EECs, *GCG* expression minimally changed and *PYY* expression dramatically increased following prolonged exposure to butyrate (117). The long-lasting effects of SCFAs on EEC L-cells suggest they may be key regulators of metabolic health and a promising dietary intervention for the treatment and management of T2DM and obesity.

### 3.2 Intracellular signaling pathways in EEC L-cells activated by bile acids

Bile acids have a functional role in lipid digestion and absorption, but also act as signaling molecules to cells lining the gastrointestinal tract. Bile acids exclusively activate two main receptors in L-cells, the cell surface G-protein coupled bile acid receptor 1 (GPBAR1, also called the membrane-type bile acid receptor, M-BAR, or the Takeda G-protein coupled receptor 5, TGR5) (118) and the nuclear transcription factor, Farnesoid-X receptor (FXR) (119–121)(**Figure 3**). Interestingly, primary bile acids preferentially activate FXR (CDCA>CA>LCA>DCA), whereas secondary bile acids are more potent endogenous ligands for GPBAR1 activation (LCA>DCA>CDCA>CA) (119). Bile acids also activate the nuclear receptors, pregnane-X receptor (PXR) (122), vitamin D receptor (VDR) (123), constitutive androstane receptor (CAR) (124), liver-X receptor (LXR) (125), and G-protein coupled sphingosine-1-phosphate receptor 2 (SIPR2) (126); however, these receptors are more selective for other endogenous and xenobiotic ligands such as steroid hormones and oxysterols.

Bile acids exert non-genomic effects through the activation of the membrane receptor, GPBAR1. Expression analysis data localizes GPBAR1 to brown adipose tissue, skeletal muscle, spleen, immune cells, gallbladder and the intestine (118, 127, 128). In the intestine, GPBAR1 is highly expressed in the ileum and colon of EECs (129, 130) and in the enteric ganglia and nerve fiber plexuses (131). GPBAR1 is localized on the basolateral face of L-cells, suggesting a mechanism is required for bile acids to be absorbed before activating the receptor (129). The transporter responsible for transporting conjugated bile acids across the epithelial layer in the small intestine is ASBT. Transport of bile acids across the epithelial layer is critical for gut hormone secretion from EECs as blocking ASBT in the terminal ileum reduced GLP-1 release (129, 132). An alternative mechanism to transport bile acids across the epithelium is required in the colon due to very low ASBT expression. Resident gut bacteria can improve bile acid permeability and potency by converting primary bile acids to secondary bile acids (132). Secondary bile acids, specifically LCA and the taurine conjugate TLCA, are the most potent stimulants of GPBAR1 activation (127, 133, 134). Multiple studies have identified bile acids as a robust trigger of GLP-1 release (28, 47, 129, 130) and bile acid-triggered GLP-1 release was diminished in a GPBAR1 knockout model (129, 133–135). GPBAR1 stimulates Gα_s_-protein coupling and increases intracellular cAMP levels through activation of adenylyl cyclase (47, 78, 129, 130). Activation of GPBAR1 in L-cells also increases membrane electrical activity *via* increased calcium current through L-type voltage-gated calcium channels (28). Bile acids can indirectly alter GLP-1 release by modulating L-cell differentiation. GPBAR1 agonists enhanced the number of GLP-1 producing L-cells in the intestinal epithelium (135).

The functional role of FXR activation is well documented (120, 136, 137). FXR regulates a multitude of genes involved in bile acid, lipid, and glucose metabolism (15). Expression of FXR is most abundant in the liver and intestine (119–121), with the highest expression levels found in the terminal ileum of EEC L-cells (45). FXR expression has also been identified in immune cells, adipose tissue, and skeletal muscle (138). FXR is a primary bile acid sensor, preferentially binding to CDCA in humans and CA to a weaker extent (119). In mice, CA is the primary ligand for FXR as mice lack CDCA (121). In the terminal ileum, activation of FXR induces expression of target genes including the small heterodimer partner (*SHP*) and fibroblast growth factor 19, *FGF19* (*Fgf15* in mice). FGF19 is released from enterocytes, transported to the liver *via* enterohepatic circulation, and binds to the tyrosine kinase receptor fibroblast growth factor receptor 4 (FGFR4) expressed in hepatocytes (139). Together, SHP and FGF19 suppress *CYP7A1*, a key gene involved in *de novo* biosynthesis of bile acids (140). The microbial ecosystem is speculated to play an important role in regulating expression of ileal FXR target genes. Under conditions of reduced gut microbiota, either germ-free or antibiotic-treated mice, elevated levels of the taurine conjugated β-muricholic acid (TβMCA) bile acid were detected (141). TβMCA acts as an FXR antagonist, resulting in reduced expression of *Fgf15* and increased *Cyp7a1* expression. Similarly, Li et al., (2013) found that reduced BSH activity diminished synthesis of secondary bile acids and inhibited FXR-induced signaling. Interestingly, the inhibition of intestinal FXR signaling altered bile acid composition in mice (142, 143) and decreased the incidence of obesity.

Activation of intestinal FXR is inhibitory to GLP-1 release in L-cells (45, 46). Trabelsi et al., (2015) determined FXR activation decreased glucose-stimulated GLP-1 secretion by blocking glycolysis, and thus glucose production in both mice and human intestinal L-cells. Similarly, Niss et al., (2020) found that inhibition of GLP-1 release by FXR is not only attributed to a downregulation in glycolysis, but also reduced glucose transport.

### 3.3 Intracellular signaling pathways in EEC L-cells activated by tryptophan, indoles and indole-derivatives

Tryptophan, the substrate for bacterial metabolism, can directly affect EEC L-cell function. In distal regions of the gastrointestinal tract, the bioavailability of digested peptides or amino acids is low as the bulk of protein digestion and absorption occurs in the small intestine before reaching the colon. Thus, the exposure of colonic EEC L-cells to tryptophan is limited. However, GLP-1 releasing L-cells in the proximal small intestine have been described (41, 144–146) and can respond to the presence of tryptophan. *In vitro*, enhanced GLP-1 release was observed in various EEC L-cell models exposed to tryptophan (147–149). However, contrary *in vivo* studies have reported a lack of stimulated GLP-1 release by intraluminal tryptophan in a perfused small intestine (150). Several G-protein coupled receptors have been implicated in EEC-sensing of tryptophan including the extracellular calcium-sensing receptor (CaSR) (151) and G-protein receptor 142 (GPR142) (149). The signaling mechanisms in EEC L-cells downstream of GPR142 activation is thought to be similar to pathways elucidated in other secretory cell types such as pancreatic β-cells (152). Both Gα_q_ and Gα_s_-proteins are thought to be recruited to increase intracellular IP_3_ and cAMP levels, respectively (152, 153). Wang et al. (2016) also demonstrated that GPR142 activation led to an increase in inositol monophosphate accumulation, thus promoting the phosphorylation of ERK. The signaling mechanisms of CaSR in EEC L-cells have not been fully characterized, but in other duodenal EEC populations activated CaSR couples to Gα_q_-protein and downstream effectors PKC and IP_3_ receptors (154).

Indoles have been shown to alter EEC L-cell function. Acute application of indole increased GLP-1 secretion by increasing calcium mobilization in L-cells (44, 155) (**Figure 4**). The mechanism of action involved inhibition of voltage-gated potassium channels, thereby causing membrane depolarization and increased mobilization of calcium (44). However, chronic indole exposure reduced GLP-1 secretion by suppressing mitochondrial adenosine triphosphate (ATP) production, thus demonstrating dual and opposing effects of indole (44). The receptor responsible for mediating the effects of indole on GLP-1 release was not identified in this study. Also, the possible actions of indoles on tryptophan-sensitive receptors CaSR and GPR142 remain to be determined.

Another regulator of indole signaling is the aryl hydrocarbon receptor (AhR). Indole, tryptamine, skatole, IAA and other indole-derivatives are ligands for AhR (71, 156). AhR is a basic helix-loop-helix (bHLH) transcription factor (157) primarily expressed in host immune cells and its activation has been shown to mediate lipid and fatty acid metabolism and intestinal homeostasis (158). Inactive AhR forms a complex with heat shock protein 90 (Hsp90), the Hsp90 chaperone p23 (P23) and X-associated protein 2 (XAP2). Ligand binding induces a conformational change and translocation of the receptor complex to the nucleus (71). Within the nucleus, gene expression is activated through binding of the AhR nuclear translocator (ARNT) protein and cis-acting AhR response elements (AhREs) in target gene promoters (157). Interestingly, indoles, IAA and IPA also activate PXR (159). As mentioned, PXR is a nuclear receptor with DNA-binding and ligand-binding domains. Activation of PXR by several products of bacterial metabolism, including secondary bile acids and indoles, suggests a convergence of gut microbiota sensing pathways. Further investigation is warranted to understand the interactions between the different activating ligands and identify common downstream effectors. These studies will provide novel insights into the mechanisms underlying gut microbiota-host interactions.

## 4 Bacterial metabolites in metabolic health

Intestinal gut composition is an important determinant of health and many studies have attributed the pathogenesis of obesity and T2DM to an altered microbial ecosystem, particularly reduced bacterial diversity (160–163). Indeed, the dysregulation of nutrient metabolism, energy homeostasis, and appetite (164), all of which occur in obese-related diseases, are associated with a colonic shift in the relative abundance of three major phyla, *Bacillota*, *Bacteroidota*, and *Verrucomicrobiota* (164–167). In earlier studies, obesity and insulin resistance were associated with an increased abundance of *Bacillota* and concomitant decrease of *Bacteroidota* in both animal (7, 168), and human studies (6, 167, 169). However, recent reports found a reduction of the *Bacillota* population in obese subjects, whereas *Bacteroidota* significantly increased (16, 170–173). Some studies have even reported no change in the abundance of the two main microbial phyla (174–177). So, the exact changes in *Bacillota* and *Bacteroidota* during metabolic disease remains unresolved and we may need to consider other patient factors such as sex (178) and diet (179). *Akkermansia¸* and its main species, *Akkermansia muciniphilia (A. muciniphilia)*, is an abundant intestinal acetate- and butyrate-producing microbe from the phylum *Verrucomicrobiota* (180–182) and is gaining interest for its protective role against T2DM and obesity (183). The presence of *A. muciniphilia* in the gut is correlated with a healthy intestine and the decline in enrichment of *A. muciniphilia* has been linked to impairments in insulin sensitivity (165) and obese-related diseases (183–185). *A. muciniphilia* improves insulin sensitivity and glucose tolerance through various anti-inflammatory and energy mechanisms (186–189).

Surgical and pharmacological interventions that improve metabolic health also alter gut microbiota populations. Patients undergoing bariatric surgery, commonly Roux-en-Y-gastric bypass (RYGB) and vertical sleeve gastrectomy (VSG), often achieve sustained weight loss and T2DM resolution (190–192). A partial restoration of healthy intestinal microbiota composition was observed six months post-bariatric surgery in morbidly-obese female participants (193).

The manipulation of gut microbiota populations may be an approach to exploit in next generation therapeutics for metabolic disease. Interestingly, fecal microbiota transplantation from an individual with a healthy gut to an individual with metabolic syndrome resulted in significantly improved insulin sensitivity, accompanied by an altered microbial composition (163). Consistent with this report, the obese phenotype in mice was found to be transmissible by transplanting the gut microbiota of conventional obese mice to normal weight germ-free mice (167, 194). Furthermore, administration of *A. muciniphilia* was safe, and improved several metabolic parameters including increased insulin activity, a reduction in insulinemia, and decreased weight status in obese, insulin-resistant patients (195). However, *A. muciniphilia* was not linked to the improved glucose homeostasis pre- or post-bariatric surgery (185). Despite these discrepancies, manipulation of gut microbiota populations or administration of the metabolites produced by beneficial gut bacteria represent a promising therapeutic approach for improving metabolic health.

### 4.1 The role of SCFAs in metabolic health

SCFAs in the gastrointestinal tract improve host gut health by increasing mucus production (196) and maintaining the intestinal gut barrier (197). SCFAs also promote crosstalk along the gut-brain axis (198) and are heavily involved in glucose and lipid metabolism (199). For example, SCFAs have been shown to stimulate the secretion of GLP-1 from intestinal L-cells (92, 95, 200) which promotes insulin release post-prandially. Similarly, butyrate attenuates insulin resistance of mice on a high-fat diet (HFD) by promoting energy expenditure (199).

Increased production of SCFAs by gut bacteria has been linked to reduced risk of obese-related chronic diseases (201). A shift in microbiome composition away from SCFA-producing bacterial species has been linked to prediabetes (173), a key step in the progression of diabetes (202). This may reflect diets consisting of high-fat and low-fiber content (203). Indeed, studies have shown that consumption of fiber- and ω-3 fatty acid-rich diets have increased levels of SCFA-producing bacteria (204, 205). The beneficial role of SCFAs was further supported by studies demonstrating increased dietary fiber intake reduced the risk of developing metabolic diseases, such as T2DM (206–210). Mechanisms proposed include direct effects on insulin sensitivity and energy expenditure (4, 199, 201, 211), improved glucose tolerance (49, 212, 213) or increased GLP-1 levels (212). Even direct delivery of SCFAs was beneficial as acute rectal infusions of sodium acetate enhanced PYY release in overweight human subjects and modulated whole-body metabolism (19–21). An increase in the abundance of acetate-producing bacteria was also found following SCFA administration (49). Therefore, supplementation of SCFAs may help to reverse gut dysbiosis as well as promote host metabolic health.

Epigenetic regulation, including HDAC-mediated mechanisms, has been linked to the development of T2DM in multiple organ systems (214). SCFAs are important regulators of gene expression through their actions as potent HDAC inhibitors (113). Reduced butyrate production led to a concomitant increase in colonic HDAC activity in a non-obese diabetic model and were associated with the increase in reactive oxidative species and alterations of colonic permeability (215). Butyrate supplementation suppressed HDAC activity in the liver of mice, leading to decreased gluconeogenesis and improved glucose homeostasis (216). This suggests that butyrate supplementation may induce epigenetic modifications that supports a healthy gut microbiota and metabolic health.

SCFAs may be important regulators of metabolic function, particularly in colonic stimulation of EECs and inhibition of HDAC activity. Harnessing the crosstalk mechanisms between resident gut bacteria and host by SCFAs may be a successful therapeutic strategy for the management of metabolic diseases in humans.

### 4.2 The role of bile acids in metabolic health

In an initial study linking cholesterol metabolism to diabetes, diabetic patients had an elevated bile acid pool size and greater excretion of bile acids in fecal samples (17). Furthermore, obese, diabetic *db/db* mice produced more bile acids leading to a larger total bile acid pool size (217), supporting the observation in humans.

The administration of bile acid sequestrants promoted insulin sensitivity in T2DM patients (218–220) and diabetic mice (221), likely by enhancing *de novo* synthesis of bile acids (222). Supporting studies have shown that in diabetic rats, intestinal sequestration of the bile acid pool improved insulin sensitivity and the mechanism involved may be mediated by GPBAR1 activation (223). Similarly, Trabelsi et al., (2015) found upon treatment with bile acid sequestrants to FXR-deficient cells, glucose tolerance improved by a GLP-1 mediated release mechanism. Direct targeting of GPBAR1 also produces positive metabolic health outcomes. In HFD-fed mice, overexpression of GPBAR1 increased secretion of GLP-1 induced insulin release, an effect that was lost in GPBAR1 deficient mice (134). Administration of oleanolic acid, the endogenous GPBAR1 agonist, ameliorated insulin sensitivity in mice upon HFD-feeding (224) and application of a selective GPBAR1 agonist, INT-777, enhanced GLP-1 secretion (133). The administration of taurocholate, the taurine conjugate of cholic acid, augmented GLP-1 release from L-cells and enhanced insulin release in humans (225). Other studies have shown that the administration of tauroursodeoxycholic acid, the taurine conjugated secondary bile acid of ursodeoxycholic acid, improved insulin sensitivity of obese humans (22) and obese mice (23). Furthermore, dual activation of FXR and GPBAR1 promotes GLP-1 release, thereby ameliorating insulin resistance (226). Thus, the receptors activated by bile acids and their metabolites present a powerful means to regulate glucose metabolism. However, intense adverse events have limited use of this approach in the clinic (227).

Dysregulation of energy utilization is often associated with obesity-related diseases (228). Activation of intestinal FXR promotes the secretion of FGF19, which could be exploited therapeutically as patients with obesity and T2DM have lower FGF19 levels (229). Administration of FGF19 to HFD-fed mice increased energy expenditure and reversed weight gain, thereby improving insulin sensitivity (230) and aided in T2DM resolution of patients following bariatric surgery (231). Interestingly, administration of a gut-biased FXR agonist protected against diet-induced weight gain while simultaneously enhancing energy expenditure (232).

Both RYGB and VSG enhanced the bile acid pool in rodents (233, 234) and humans (235, 236). Studies have shown that HFD-fed mice subjected to a bile diversion procedure increased the abundance of circulating bile acids (141, 237, 238) and improved glucose homeostasis (142). This was confirmed by the loss of significant weight reduction in FXR knockout mice on a HFD following VSG (239). However, increased abundance of bile acids has been reported in obese individuals with T2DM (240). Further, Jahansouz et al., (241) found a hypocaloric diet, mimicking weight loss, reduced the abundance of unconjugated bile acids. Bile acid signaling is involved in energy expenditure and glucose control following bariatric surgery in obese mice (239, 242) but further study is needed to define the role of bile acid signaling after bariatric surgery in humans.

### 4.3 The role of indoles and indole-derivatives in metabolic health

Amino acid metabolism has been linked to metabolic health for decades; however, the involvement of metabolic products from bacterial amino acid metabolism in host metabolic health has recently become of interest. Fecal concentrations of indole, IAA and tryptamine were significantly reduced in mice fed a HFD compared with chow-fed mice (18, 243). A corresponding reduction in the concentration of indole-derivatives was reported in the feces of clinically obese (BMI>30) or T2DM human participants. Similar reductions in serum levels of indole-derivatives were reported in obese participants compared with non-obese controls (244). Following RYGB bariatric surgery, there was a significant improvement in glucose tolerance in T2DM subjects which coincided with an increase in IPA and tryptamine levels (245). Other retrospective studies have demonstrated that higher IPA levels were associated with lower risk of developing T2DM (70, 245).

Classical studies investigating the benefits of tryptophan consumption on metabolic disease progression may be attributed to bacterial-derived tryptophan metabolites. Ingesting tryptophan-enriched diets lowered the risk of developing obesity and T2DM in humans (246) and suppressed hyperglycemia and weight gain in animal models (247–249). Some of the mechanisms proposed for tryptophan-suppressed hyperglycemia include reduced insulin production and protection of pancreatic β-cells in diabetic rats (247) or inhibition of gluconeogenesis in rats and guinea pigs (248). The gut bacteria-derived tryptophan metabolite IPA was also associated with improved β-cell function (250) and rats fed an IPA-rich diet had significantly reduced fasting glucose levels (24). Furthermore, lower serum IPA levels were the most relevant indicators of early-onset T2DM (251) and body weight changes in obese rats (252). Recent studies have evaluated serum IPA levels as a risk biomarker for developing T2DM (250) or obesity (245) in humans. However, increased levels of tryptophan in the blood has been proposed as a predictor of increased risk of developing T2DM (253). Perhaps, this discrepancy between tryptophan and indolic metabolites in predicting metabolic disease states is due to the diverging metabolic pathways involved in the degradation of tryptophan. Tryptophan metabolites derived from the kynurenine pathway and IAA were found to be positively associated with T2DM risk (254). The kynurenine pathway exclusively involves host metabolism of tryptophan and excess kynurenine-metabolites have been associated with neurotoxicity and inflammation (255), while IAA is an intermediate of bacterial tryptophan metabolism and may be indicative of disrupted indole biosynthesis. As previously mentioned, the exclusive microbial-derived metabolite IPA has antidiabetic properties (24, 250). Therefore, the diversion of tryptophan metabolism to bacterial production of indoles or IPA has proven benefits in enhancing a healthy metabolic state.

Indole has been previously studied as an intercellular signaling molecule within the gut microbiota ecosystem. The capacity of indoles to stimulate GLP-1 releasing L-cells (44) has recently become of interest as a potential therapeutic target to regulate metabolic dysfunction (250). Indole-stimulated GLP-1 release can trigger the systemic effects of GLP-1 including enhanced insulin secretion, reduction in appetite and slowing of gastric emptying (29). Another possible mechanism by which indoles may regulate metabolic function could be through the regulation of intestinal microbial populations. Indoles directly affect bacterial functions associated with protection and host colonization (256). Since the relative abundance of several intestinal bacteria is associated with metabolic disease states, the role of indoles in determining the composition of the gut microbiota may contribute to the beneficial outcomes of bacterial indole production.

Indoles and other indole-derived metabolites produced by gut bacteria have significant physiological effects which may be exploited in future therapeutics. However, the risks associated with production of toxic by-products such as indoxyl sulfate will have to be carefully considered. Engineering enterobacteria that favor production of non-toxic indolic metabolites or the development of synthetic indole analogues that bypass first-pass metabolism are potential approaches to explore in designing future gut microbiota-based therapies. Herbal medicines may provide a source of inspiration to design structural analogues of indoles to treat metabolic disease. Indole alkaloids, which are bioactive compounds isolated from plants, have been found to inhibit dipeptidyl peptidase IV (DPP-IV) (257), the enzyme responsible for the inactivation of GLP-1. Therefore, indole alkaloids increase the half-life of the gut hormone responsible for enhancing insulin release post-prandially. Pharmacological strategies based on plant extracts containing indole alkaloids are effective in treating diabetic rats (258, 259) but their effectiveness and safety in human clinical trials remain to be determined. The direct effects of indole alkaloids on GLP-1 release are unknown, raising the possibility of these plant-based therapies improving glucose homeostasis through multiple synergistic pathways. Novel therapeutic approaches could also exploit indole signaling pathways. Recent studies have shown that targeting AhR by indoles may be able to ameliorate diabetes. Supplementation of indole-3-carbinol (I3C), an endogenous ligand of AhR, increased expression of AhR in the intestine (260) and promoted weight loss of HFD-fed obese mice and improved glucose tolerance (261). The involvement of AhR signaling in reducing proinflammatory responses (262, 263) could promote additional improvements in the treatment of metabolic diseases.

### 4.4 The role of other bacterial metabolites in metabolic health

We have focused our review on the main metabolites produced by gut bacteria, namely short-chain fatty acids, secondary bile acids and indoles. However, other bacterial metabolites have also been implicated in host metabolic health. Bacterial fermentation of other dietary amino acids can give rise to several active metabolic compounds. For instance, p-Cresol is produced following tyrosine or phenylalanine degradation by gut bacteria. Serum concentration of p-Cresol is negatively correlated with T2DM and administration of p-Cresol reduced body weight, improved glucose homeostasis and β-cell function in HFD-fed mice (264). Despite these promising results, p-Cresol as a therapy for metabolic disease is limited as oral routes of administration are contraindicated due to sulfation by host cells to the nephrotoxic metabolite p-Cresol sulfate (264, 265). In addition, p-Cresol itself is a volatile compound that induces detrimental neurological, liver and respiratory effects at high concentrations (266). Imidazole propionate (IMP) is another amino acid-derived metabolite, produced by gut bacterial metabolism of histidine. Human subjects with prediabetes or T2DM have increased serum IMP levels compared with healthy individuals and administration of IMP to mice impairs glucose tolerance and disrupts insulin receptor signaling pathways (267, 268). Similarly, inosine, a purine metabolite involved in nucleotides and nucleic acids, is positively correlated with T2DM risk (269). Both IMP and inosine may be potential biomarkers to identify metabolite changes by the gut microbiota or may be exploited to uncover future treatments. More work is warranted to reconcile the changes that occur to the microbial composition and the mechanism by which these metabolites act to increase metabolic disease risk.

## 5 Conclusion

The gut microbiota has emerged as a pivotal regulator of GLP-1 releasing L-cells. Various approaches to treat obese-related diseases have been of interest for decades, although novel therapeutic strategies are urgently needed to treat a growing patient population. Approaches such as combining existing therapies in order to further enhance weight loss with fewer side effects or targeting the gut microbiota are currently in use. Altering gut bacterial populations with pre- or probiotics is a popular strategy to exploit this important relationship and restore deficiencies of nutrient and energy homeostasis observed in obesity-associated diseases. More work is needed to understand the precise cellular mechanisms that govern the bidirectional communication between the gut microbiota and EECs. Targeting common downstream effectors of converging signaling pathways recruited following bacterial metabolite receptor activation may be a feasible strategy as well. Due to the increasing incidence of metabolic disease, understanding the symbiotic relationship between gut bacteria and host cellular function, will provide greater clarity for the development of novel therapeutic strategies for the treatment of obese-related diseases.

## Author contributions

KM and VL wrote and reviewed the manuscript. Both authors contributed to the article and approved the submitted version.

## Glossary

**Table:**

AhR	aryl hydrocarbon receptor
ASBT	apical sodium-dependent bile acid transporter
ATP	adenosine triphosphate
BSH	bile salt hydrolase
C2/C3/C4	acetate/propionate/butyrate
CA	cholic acid
cAMP	cyclic adenosine monophosphate
CAR	constitutive androstane receptor
CaSR	calcium-sensing receptor
CDCA	chenodeoxycholic acid
CYP450	cytochrome P450
DCA	deoxycholic acid
EEC	enteroendocrine cell
ERK	extracellular signal-regulated kinase
FFA2/3	free fatty acid receptor 2 or 3
*Ffar/FFAR*	mouse/human free fatty acid receptor gene
FGF15/FGF19	fibroblast growth factor 15 in mouse/fibroblast growth factor 19 in human
*Fgf15/FGF19*	fibroblast growth factor 15 gene in mouse/fibroblast growth factor 19 gene in human
FGFR4	fibroblast growth factor receptor 4
FXR	Farnesoid-X receptor
*Gcg/GCG*	mouse/human glucagon gene
GLP-1	glucagon-like peptide-1
GPBAR1	G-protein coupled bile acid receptor (also called the M-BAR, membrane type bile acid receptor, TGR5, Takeda G-protein-coupled receptor 5)
GPCR	G-protein coupled receptor
GPR109A	G-protein coupled receptor 109A
GPR142	G-protein coupled receptor 142
*GPR142*	G-protein coupled receptor 142 gene
HCAR2	hydroxycarboxylic acid receptor 2
HDACs	histone deacetylases
HFD	high-fat diet
IAA	indole-3-acetic acid
IAld	indole-3-aldehyde
IMP	imidazole propionate
IP_3_	inositol triphosphate
IPA	indole-3-propionic acid
IPyA	indole-3-pyruvic acid
LCA/TLCA	lithocholic acid/taurine conjugate of lithocholic acid
LXR	liver-X receptor
MCA/ω-MCA/TβMCA	muricholic acid/omega-muricholic acid/taurine conjugate β-muricholic acid
MCTs	monocarboxylate transporters
OR51E1/OR51E2	human olfactory subfamily (Olfr558/Olfr78 in mouse)
PXR	pregnane-X receptor
PYY	peptide YY
RYGB	Roux-en-Y-gastric bypass
SCFAs	short-chain fatty acids
SHP	small heterodimer partner
*SHP*	small heterodimer partner gene
SIPR2	G-protein coupled sphingosine-1-phosphate receptor 2
SMCTs	sodium-dependent monocarboxylate transporters
T2DM	type 2 diabetes mellitus
VDR	vitamin D receptor
VSG	vertical sleeve gastrectomy
